# The Future of Virtual Care for Older Ethnic Adults Beyond the COVID-19 Pandemic

**DOI:** 10.2196/29876

**Published:** 2022-01-07

**Authors:** Quynh Pham, Noor El-Dassouki, Raima Lohani, Aravinth Jebanesan, Karen Young

**Affiliations:** 1 Centre for Global eHealth Innovation Techna Institute University Health Network Toronto, ON Canada; 2 Institute of Health Policy, Management and Evaluation Dalla Lana School of Public Health University of Toronto Toronto, ON Canada; 3 Telfer School of Management University of Ottawa Ottawa, ON Canada; 4 Global Health Office Faculty of Health Science McMaster University Hamilton, ON Canada

**Keywords:** virtual care, digital health, health equity, cultural equity, chronic disease, caregivers, ethnocultural minority, older adults, ethnicity, ethnic patients, technology-mediated care, equity, diversity, family

## Abstract

The COVID-19 pandemic has fundamentally changed how Canadians access health care. Although it is undeniable that the rapid adoption of virtual care has played a critical role in reducing viral transmission, the gap in equitable access to virtual care remains pervasive for Canada’s aging and ethnocultural minority communities. Existing virtual care solutions are designed for the English-speaking, health-literate, and tech-savvy patient population, excluding older ethnic adults who often do not see themselves reflected in these identities. In acknowledging the permanency of virtual care brought on by the pandemic, we have a collective responsibility to co-design new models that serve our older ethnic patients who have been historically marginalized by the status quo. Building on existing foundations of caregiving within ethnocultural minority communities, one viable strategy to realize culturally equitable virtual care may be to engage the highly motivated and skilled family caregivers of older ethnic adults as partners in the technology-mediated management of their chronic disease. The time is now to build a model of shared virtual care that embraces Canada’s diverse cultures, while also providing its older ethnic adults with access to health innovations in partnership with equally invested family caregivers who have their health at heart.

## Virtual Care in a Postpandemic World

The COVID-19 pandemic has fundamentally changed how all Canadians access health care. As initial media coverage showed unsettling scenes of Canada becoming overwhelmed by this new virus, we felt a sense of unease about our own health and the new ways in which we would receive care. With our health care systems stretched and strained to reduce the devastation wreaked by the pandemic, virtual care solutions were rapidly adopted to maintain continuity of care and deliver essential services. Yet in looking deeper at how virtual care became the new standard of care during the pandemic, we know now that this technological transformation did not always benefit those most in need.

Virtual care is defined as “any interaction between patients and/or members of their circle of care, occurring remotely, using any forms of communication or information technologies, with the aim of facilitating or maximizing the quality and effectiveness of patient care” [1]. The first wave of the COVID-19 pandemic forced a sharp decline in in-person primary care visits in Canada’s most populous province of Ontario as phone and video consultations became the de facto means of accessing care [2]. The second wave of the COVID-19 pandemic saw an even greater shift to virtual care delivery across diverse health care settings such as physiotherapy and postoperative care [3]. Although it is undeniable that virtual care has played a critical role in reducing viral transmission, the future of health care cannot simply be a matter of transitioning current models of care to virtual settings. Sustaining virtual care beyond the pandemic requires us to thoughtfully consider the emerging needs of our society. The Canadian population is growing older, and at the same time, more ethnically diverse. When we look at Canada’s growing demographic of aging and ethnically diverse adults, the risk of inequity is stark. First, older adults aged 65 years or older comprise 18% of the total population and 9% of the visible minority population [4,5]. Second, visible minority older adults are the fastest growing demographic in Canada [6]. Finally, the impact of chronic disease among older adults places a growing burden on our health care system: over 70% of older adults have been diagnosed with at least one of the 10 most common chronic diseases [7,8]. Without a shared strategy to provide equitable access to health care for this emerging population, virtual care runs the risk of exacerbating existing health care inequities for older ethnic adults living with chronic disease [9,10].

In this viewpoint, we refer to the ethnic groups that make up a minority of the Canadian population according to census data and the cultures which characterize them as “ethnocultural minority” communities [11]. Ethnicity comprises the shared aspects of belonging to a group, such as language, ancestry, nationality, and values [12]. The social practices, behavioral norms, and physical expressions of societies—such as traditions, customs, and ideologies—form their culture [13]. Our use of the term “ethnocultural” captures both the shared group aspects and social practices that define ethnicity and culture respectively. In using this terminology, we aim to highlight the ethnic and cultural aspects of caring as factors that influence how patients are able to access virtual care but are often not considered in the design of virtual care platforms.

Early findings on the spread of virtual care during the pandemic in the United States have revealed that older adults, those with limited English proficiency, and those from ethnocultural minority communities have reduced access to virtual care services [10]. Promising work has been done at the intersection of aging, technology, and diversity to improve equity and access to virtual care; solutions include involving patients in technology co-design, increasing access to language interpreters, developing training materials in collaboration with community-based organizations, equipping community centers with the devices and space to receive virtual care, and offering low-tech care options such as telephone visits to improve access [14,15]. However, existing virtual care solutions are often designed for the English-speaking, health-literate, and tech-savvy patient population, and are informed by a biomedical model of health and illness. Older ethnic adults often do not see themselves reflected in these identities and, as such, continue to be excluded from receiving virtual care. Issuing these communities an ultimatum to successfully learn complex technological and self-management behaviors in order to receive care will likely result in even greater isolation and heighten their existing struggles to safely age in place. This feat can be insurmountable in the best of times and is even more overwhelming during a global pandemic when these communities are already experiencing greater health and economic needs [16-19]. Current virtual care models ultimately fail to acknowledge the reality that the same older ethnic adults who are challenged to adopt virtual care are also more likely to have complex health needs and greater difficulties accessing health care [20]. For a population that already experiences deeply rooted health, social, and economic inequities, the inability to access virtual care nearly guarantees that older ethnic adults will struggle to receive *any* care from increasingly digital-first health systems in a postpandemic world. We believe a new model of virtual care that rejects burdening older ethnic adults with complex requirements in order to access care is desperately needed. One viable strategy to realize culturally equitable virtual care may be to engage the highly motivated and skilled family caregivers of older ethnic adults as partners in the technology-mediated management of their chronic disease.

## The Opportunity for Family Caregivers to Become Virtual Care Partners

Virtual care is here to stay. In acknowledging this new reality, we have a rare opportunity to engage older ethnic adults in virtual care by building on the values that are deeply embedded in their collectivist communities [21]. Despite nuances in how caregiving is defined across cultures, there is a common perception that caring for one’s family is a natural and expected part of life [22]. Across many Asian cultures, familial kinship and filial piety represent a deep commitment to caring for aging parents [21]. Although familial kinship places greater value on sharing caregiving responsibilities across family members, filial piety focuses specifically on the parent-child relationship and the practice of caring for one’s parents [21,23]. Both familial kinship and filial piety emphasize the value of intergenerational relationships and family-centered approaches to caregiving, which see adult children heavily involved in the role of caring for their ailing parents [23]. We can build on the existing foundations of caregiving within ethnocultural minority communities to establish older ethnic adults and their family caregivers as true partners in virtual care.

Acting on the opportunity presented by this model of care requires us to first understand the intrinsic strengths of family caregiving. Family caregivers provide comprehensive and critical assistance to older ethnic adults navigating the health care system [24]. Although this role can be assumed by partners, siblings, grandchildren, extended family, and friends, the vast majority of caregivers in Ontario are adult children [25]. Through a lifetime of shared experiences, adult children bring an invaluable wealth of knowledge about their parents’ health, social, economic, and supportive needs. Their linguistic and cultural relation to their parents affords them some jurisdiction to make joint decisions on how to manage health and illness [26]. These values of familial kinship and filial piety, the cultural embeddedness of caregiving, and the ability to inform health care preferences and practices can empower adult children to not only care for their parents, but also to see the value of participating in chronic disease comanagement using virtual care [21-23,27-31]. Many adult child caregivers are also highly educated and have strong digital and health literacy skills [32-34]. Recent census data examining second-generation Chinese and South Asian Canadians—communities that place high value on filial piety—revealed that these groups are generally highly educated and working in advanced information technology occupations [35]. The adult children of older ethnic patients thus present as an extremely valuable resource with skills that will allow them to be the index user of virtual care technologies in the patient-caregiver partnership, thereby removing the burden on their parents to adopt technology [32].

Leveraging this opportunity to comanage chronic diseases through virtual care also requires us to carefully consider the challenges and implications of family caregiving. Caring for a sick family member is psychologically, physically, socially, and financially demanding; experiences of caregiver burnout are unfortunately common [36-39]. The cost of caring for an older family member and witnessing their loss of independence and functional decline can manifest as negative feelings of loss, hostility, and anxiety among family caregivers and strain relationships [40-42]. Caregivers of ethnocultural minority communities also exhibit higher levels of involvement in care activities compared to other ethnic groups, which can further impede their quality of life [43]. In particular, the cultural norms of many ethnocultural minority communities often place the responsibility of caregiving on wives, daughters, and daughters-in-law [23]. Virtual models of shared care should be designed in a way that reduces this gendered burden and removes the need for women to make personal sacrifices to fulfill their filial responsibilities [44].

Significant privacy implications must be considered when operationalizing a model of shared virtual care. Although there are many benefits to sharing personal health information in this context, patients and their caregivers should be informed of the risks of granting shared access. Having caregivers be privy to previously unknown and oftentimes sensitive information can lead to undesired changes to the parent-child relationship and an unintended loss of patient autonomy [45]. For instance, gaining access to personal information can lead caregivers to dominate care decisions and communication with providers, thereby reducing the patient’s participation in, and control of, their own care [46]. Establishing routine communication exercises that revisit roles and responsibilities around shared decision-making and care actions, and creating processes that ensure active patient engagement, can help partners to better navigate their shared roles.

## Reimagining Virtual Care to Be Culturally Equitable for Older Ethnic Adults

The future of virtual care for older ethnic adults beyond the COVID-19 pandemic will require a reimagining of the ways in which ethnocultural minority communities access and receive care. Virtual care models that formally engage caregivers have shown promise in improving access to primary and chronic care for older ethnic adults. Latulipe and colleagues [47,48] recently showed that creating caregiver proxy user accounts in patient health portals can allow patients with limited English language proficiency to fully benefit from technologies they would be unable to access otherwise. Piette and colleagues [49-52] have led a decade’s worth of formative and definitive trials to study the effectiveness of the CarePartner program and its implementations for heart failure, cancer, diabetes, and mental health. Patients in the CarePartner program complete automated symptom assessments through an interactive voice response (IVR) system and receive tailored health and behavioral advice on how to manage their symptoms. Their caregiver receives a structured report automatically via email with information about the patient’s health and what they can do to help. The program has demonstrated significant improvements in symptom management, medication adherence, health service utilization, and emotional coping, with patient-partner dyads reporting improved frequency and quality of communication [51-54]. It is worth noting that the CarePartner Program maintained its effectiveness when deployed in Bolivia to support the dyadic management of diabetes and hypertension [49]. Findings from the randomized trial comparing IVR alone or with automated feedback sent to a caregiver after each IVR assessment showed that caregiver feedback increased engagement with IVR most significantly among patients of Indigenous ethnicity and those with low functional health literacy [55].

**Figure 1:**
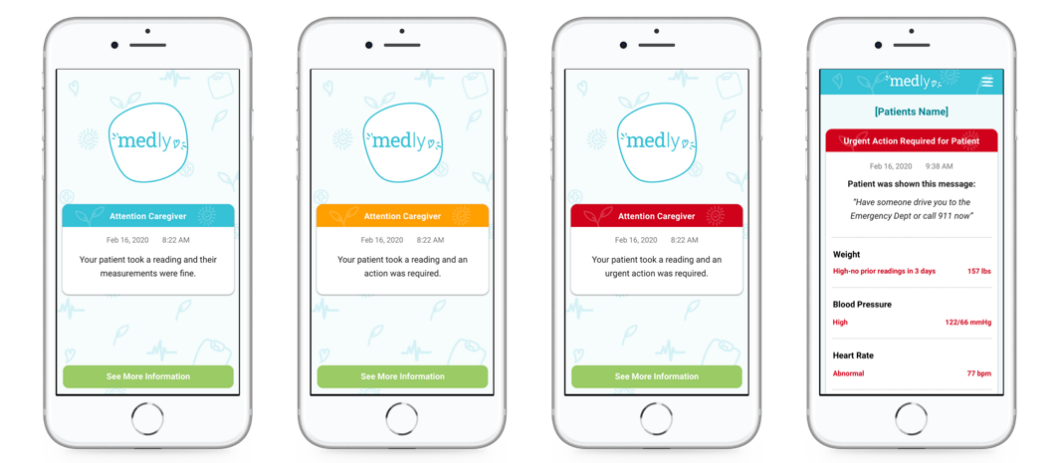
Medly Caregiver Live Report screenshots displaying patient partner health status and data generated on the Medly patient app.

Motivated by the successes of dyadic virtual care programs, our team [56] has started to formalize caregiver inclusion in the service design and delivery of our virtual clinics for heart failure and cancer survivorship. Our Medly Clinic [57] supports remote heart failure monitoring [58-61], while our Ned Clinic [62] enables asynchronous prostate cancer survivorship care [63,64]. Both virtual clinics have historically excluded patients who were not comfortable using technology, did not have an email address, and had limited English proficiency; we previously assumed that these factors would challenge patients to use clinic services as intended and derive benefit. The onset of the COVID-19 pandemic created urgency to eliminate barriers to the Medly and Ned clinics as they enabled the provision of services like medication titration and follow-up visits that were paused due to institutional lockdown orders. As a result of increased patient demand for these services, both clinics began enrolling a growing proportion of older ethnic adult patients who were accompanied by their family caregivers and relied on them to adopt the clinic technology. This off-label dyadic approach to onboarding patients who would otherwise not qualify for virtual clinic services brought to light a unique opportunity to formally adapt the clinics for diverse cultural groups. We have since developed Medly Caregiver Live Reports to enable caregivers to view their partner’s data generated on the Medly patient app (Figure 1). In parallel to this effort, we drafted new standard operating procedures to formally enroll patient-caregiver dyads into the Ned Clinic and support their shared use of the platform. Our Ned Partners service includes expanded recruitment and onboarding materials, a tips sheet and informal agreement for dyads to align on the challenges of survivorship comanagement, and adapted workflows to accommodate changes in their involvement in the clinic and ownership of care tasks over time (Multimedia Appendix 1).

We believe that Canada’s virtual care road map requires systemic reform to improve the health and social outcomes of its ethnocultural minority communities. By 2031, 32% of Canada’s population is projected to belong to a visible minority group [65]. As this population ages, they deserve to receive virtual care that they perceive to be acceptable, appropriate, and aligned with their cultural beliefs. We have a collective responsibility to co-design new models of virtual care that reflect these ideals and deliver measurable and sustained impact. Only then can virtual care transition from an exclusive service to an equitable standard that serves those patients who have been historically marginalized by the status quo. The time is now to build a model of shared virtual care that embraces Canada’s diverse cultures and provides its older ethnic adults with access to health innovations in partnership with equally invested family caregivers who have their health at heart.

## Appendix

Multimedia Appendix 1 Ned care partners tip sheet.

## Abbreviations

IVR: interactive voice response
