# Supplementary material for: The Future of Virtual Care for Older Ethnic Adults Beyond the COVID-19 Pandemic
Source: J Med Internet Res. 2022 Jan 7;24(1):e29876. doi: 10.2196/29876 (PMC8783290; doi:10.2196/29876)
Supplement: Multimedia Appendix 1 [file jmir_v24i1e29876_app1.pdf]

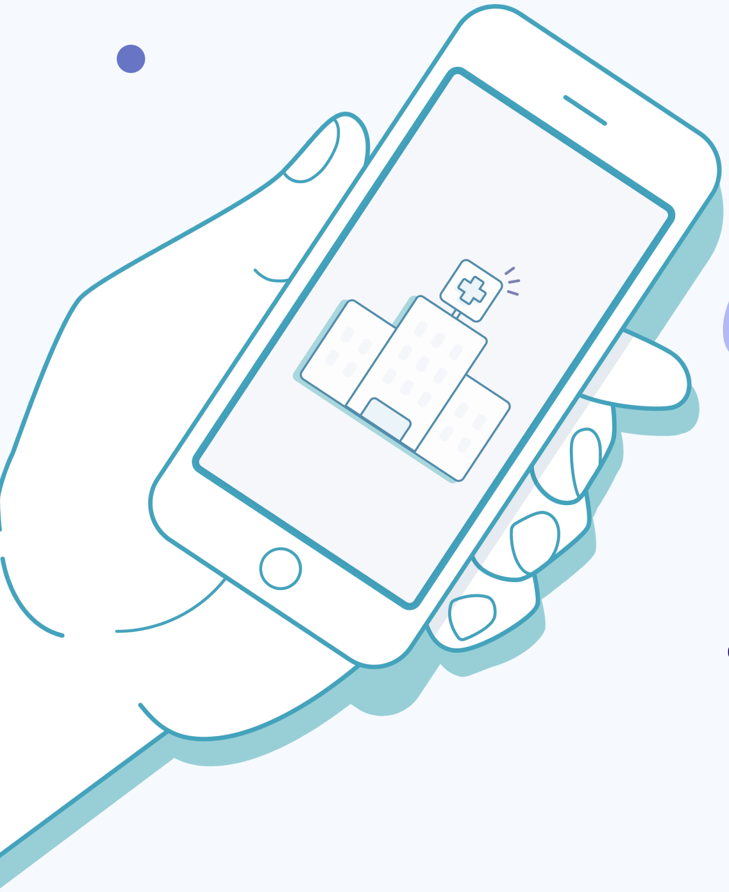

Ned

Tip Sheet for Care Partners

Using *Ned* as Care Partners

Being partners in managing care can be a rewarding and challenging experience as you both work together towards a common goal: having the best possible survivorship experience. Having a care partner can improve a patient's quality of life, including their spiritual wellbeing and mental health.[1] Supporting a patient in their care can improve the caregiver's personal growth, perceived health, and overall well-being.[2]

Review the following checklist together to strengthen your team as care partners: [3]

- ❏ Discuss both your needs and determine what you both are able and unable to do within your roles as a patient or caregiver
- ❏ Decide who will be responsible for the different aspects of care, such as printing or sending lab requisitions to the lab. This will help avoid confusion between you and your care partner.
- ❏ Work together to decide how openly you wish to communicate with one another about symptoms and your relationship
- ❏ Revisit your roles as care partners and level of communication on a regular basis, such as every 2 months
- ❏ Solve problems and plan for the future together
- ❏ Focus on short-term and achievable goals, such as substituting pop for water
- ❏ Recognize each other's accomplishments - co-managing care is difficult!
- ❏ Avoid blame and do your best to actively listen to each other
- ❏ Understand that there may be changes to your personal relationship:
    - ❏ As the patient, be aware that your care partner will have access to your personal health information and this may result in feelings of loss of control. Remember - this is a normal feeling to have as you both work through your partnership roles.
    - ❏ As the caregiver, recognize that you will have access to information you may not have had before and this may affect how you perceive your personal relationship.
- ❏ Remember that no team is perfect - honest, open, and clear communication will foster a more unified experience as you share care tasks

---

Completing the Wellness Survey

The goal of the Wellness Survey is to gather information on symptoms the patient may be experiencing. Some of the survey questions cover sensitive topics that you may find uncomfortable to discuss with your care partner.

As care partners, consider doing the following to ensure you receive the most appropriate care:

- ❏ Complete the Wellness Survey honestly so your doctor can help you manage your symptoms and track your progress most effectively.
- ❏ Approximately 1 week before your follow-up tasks are due, verify your responses to the Wellness Survey with one another to make sure the health information you will be submitting is correct. As a caregiver, try not to assume what the patient is experiencing.
- ❏ Show the patient how to complete the Wellness Surveys independently if they wish to do so.
- ❏ If applicable, care partners may work together to translate the Wellness Survey into the patient's native language. You may wish to ask the Ned Coordinator during your Welcome Visit or by emailing [redacted] about using a cultural translator to assist with translating the survey.

Virtual Visits

Your doctor may sometimes schedule a virtual (video or phone) visit to discuss your progress with you. Keep the following in mind to make the most out of your virtual visits:

- Before your appointment, create a list of any questions or concerns you have. Bring them up at the end of your appointment if they were not addressed during the appointment.
- The visit will be most effective if one speaker is speaking at a time. Wait for the doctor to prompt either one of you for questions or input before speaking.
- To indicate a desire to speak during a video appointment, you may wish to use the "raise hand" function or type in the chat box.

Other Things to Keep in Mind

- The patient must provide their consent before any account changes, like email or password changes, are made
- *Ned* Clinic staff cannot control who you share password and security question information with
- Depending on your past experiences as care partners, using *Ned* may cause the caregiver to feel a change in their level of responsibility with managing the patient's care
- At any point, if the patient or care partner wishes to disengage as a team and the patient wishes to continue using *Ned* independently, please contact [redacted].

References

1. Hu, Y., Liu, T. & Li, F. Association between dyadic interventions and outcomes in cancer patients: a meta-analysis. *Support. Care Cancer* **27**, 745–761 (2019).

2. Jones, P. S., Winslow, B. W., Lee, J. W., Burns, M. & Zhang, X. E. Development of a caregiver empowerment model to promote positive outcomes. *J. Fam. Nurs.* **17**, 11–28 (2011).

3. Li, Q. & Loke, A. Y. A literature review on the mutual impact of the spousal caregiver–cancer patients dyads: 'Communication', 'reciprocal influence', and 'caregiver–patient congruence'. *Eur. J. Oncol. Nurs.* **18**, 58–65 (2014).
